# Body fatness and breast cancer risk in relation to phosphorylated mTOR expression in a sample of predominately Black women

**DOI:** 10.1186/s13058-021-01458-z

**Published:** 2021-07-30

**Authors:** Ting-Yuan David Cheng, Angela R. Omilian, Song Yao, Weizhou Zhang, Susmita Datta, Wiam Bshara, Rochelle Payne Ondracek, Warren Davis, Song Liu, Chi-Chen Hong, Elisa V. Bandera, Thaer Khoury, Christine B. Ambrosone

**Affiliations:** 1 Department of Epidemiology, University of Florida, 2004 Mowry Road, 4th Floor, PO Box 100231, Gainesville, FL 32610 USA; 2 Department of Cancer Prevention and Control, Roswell Park Comprehensive Cancer Center, Buffalo, NY USA; 3 Department of Pathology, Immunology and Laboratory Medicine, University of Florida, Gainesville, FL USA; 4 Department of Biostatistics, University of Florida, Gainesville, FL USA; 5 Department of Pathology & Laboratory Medicine, Roswell Park Comprehensive Cancer Center, Buffalo, NY USA; 6 Department of Biostatistics and Bioinformatics, Roswell Park Comprehensive Cancer Center, NY Buffalo, USA; 7 Cancer Epidemiology and Health Outcomes, Rutgers Cancer Institute of New Jersey, The State University of New Jersey, New Brunswick, NJ USA

**Keywords:** Breast cancer, Mechanistic target of rapamycin, African American/Black women, Body fatness, Case-control study

## Abstract

**Background:**

The mechanistic target of rapamycin (mTOR) pathway promoted by positive energy imbalance and insulin-like growth factors can be a mechanism by which obesity influences breast cancer risk. We evaluated the associations of body fatness with the risk of breast cancer varied with phosphorylated (p)-mTOR protein expression, an indication of the pathway activation.

**Methods:**

Women with newly diagnosed breast cancer (*n* = 715; 574 [80%] Black and 141 [20%] White) and non-cancer controls (*n* = 1983; 1280 [64%] Black and 713 [36%] White) were selected from the Women’s Circle of Health Study. Surgical tumor samples among the cases were immunostained for p-mTOR (Ser2448) and classified as p-mTOR-overexpressed, if the expression level ≥ 75th percentile, or p-mTOR-negative/low otherwise. Anthropometrics were measured by trained staff, and body composition was determined by bioelectrical impedance analysis. Odds ratios (ORs) of p-mTOR-overexpressed tumors and p-mTOR-negative/low tumors compared to controls were estimated using polytomous logistic regression. The differences in the associations by the p-mTOR expression status were assessed by tests for heterogeneity.

**Results:**

Cases with p-mTOR-overexpressed tumors, but not cases with p-mTOR-negative/low tumors, compared to controls were more likely to have higher body mass index (BMI), percent body fat, and fat mass index (*P*-heterogeneity < 0.05), although the OR estimates were not significant. For the measurement of central adiposity, cases with p-mTOR overexpressed tumors had a higher odds of being at the Q3 (OR = 2.52, 95% CI = 1.46 to 4.34) and Q4 (OR = 1.99, 95% CI = 1.12 to 3.50) of waist circumference (WC) compared to controls. Similarly, cases with p-mTOR overexpressed tumors had a higher odds of being at the Q3 (OR = 1.82, 95% CI = 1.11 to 2.98) and Q4 (OR = 1.81, 95% CI = 1.11 to 2.98) of WHR compared to controls. These associations of WC and waist-to-hip ratio (WHR) did not differ by tumor p-mTOR status (*P*-heterogeneity = 0.27 and 0.48, respectively).

**Conclusions:**

Our findings suggest that in this population composed of predominately Black women, body fatness is associated with breast cancer differently for p-mTOR overexpression and p-mTOR negative/low expression. Whether mTOR plays a role in the obesity and breast cancer association warrants confirmation by prospective studies.

**Supplementary Information:**

The online version contains supplementary material available at 10.1186/s13058-021-01458-z.

## Introduction

The underlying mechanisms between obesity and breast cancer development are not completely understood and may differ by cancer subtype and body size measurement. A widely accepted model is that, in postmenopausal women, obesity increases circulating estrogen levels through their aromatization in adipose tissue and promotes estrogen receptor–positive (ER+) breast cancer [1]. This model is supported by the positive association between obesity vs. normal weight (body mass index [BMI], calculated as weight in kilograms divided by height in meters squared, ≥ 30 vs. < 25, respectively) and ER+ breast cancer risk in postmenopausal women [2–4]. In the African American Breast Cancer Epidemiology and Risk (AMBER) Consortium, a similar association between BMI and ER+ breast cancer was also observed in Black women. However, the consortium data also showed that waist-to-hip ratio (WHR) was associated with increased risk of ER+ or ER-negative (ER–) breast cancer in either premenopausal or postmenopausal Black women [5]. This finding on the WHR and ER– tumor association suggests that other mechanisms associated with central obesity besides the estrogen pathway may play a role in breast cancer development.

The mechanistic target of rapamycin (mTOR) pathway has been suggested as a mechanism underlying obesity and breast cancer development [6]. This pathway is directly promoted by positive energy imbalance and insulin-like growth factors (IGFs) [7, 8], and higher vs. lower concentrations of IGF-1 are associated with an increased risk of breast cancer [9, 10]. Activated mTOR stimulates translation and inhibits autophagy, and lower mTOR activity decreases tumor incidence in animal models [11]. We previously reported that genetic variants in the mTOR pathway, including *MTOR*, were associated with increased risk of breast cancer [12, 13], and there was a potential gene-environment interaction that the association for a variant was stronger in obese vs. normal-weight women [13]. The mTOR pathway relies on protein phosphorylation for signaling [8], and phosphorylated (p) mTOR protein expression levels in breast tumors were strongly associated with BMI and body fatness [14]. However, research has not compared breast cancer cases of high levels of p-mTOR expression with healthy controls to reveal the etiologic role of mTOR as a mechanism of obesity and breast cancer development. Understanding such a mechanism is important to inform preventive strategies for obesity-related breast cancer.

Here, with a case-control design, we evaluated the associations of body fatness, measured by BMI, waist circumference (WC), WHR, percent body fat, and fat mass index, with breast cancer by the expression level of p-mTOR, which is evidence of mTOR activation [14, 15]. We hypothesized that cases with p-mTOR overexpression tumors, but not those with p-mTOR negative/low expression tumors, would have increased odds of having higher rather than lower levels of body fatness compared to controls.

## Methods

### Study participants

This study followed the Strengthening the Reporting of Observational Studies in Epidemiology (STROBE) reporting guideline. Study participants were women recruited between 2001 and 2015 for the Women’s Circle of Health Study (WCHS), a multisite case-control study conducted in New York City and 10 counties in eastern New Jersey. Details on study recruitment have been described elsewhere [16, 17]. All participants provided written informed consent. The protocol was approved by all relevant institutional review boards. In brief, the cases included patients who self-identified as Black or as White women, were 20–75 years of age with no previous history of cancer other than nonmelanoma skin cancer, and were within 9 months of having received a diagnosis of primary, histologically confirmed, invasive breast cancer or ductal carcinoma in situ (DCIS). Controls had the same inclusion criteria but no history of cancer and were identified during the same period. Controls were obtained by random digit dialing and frequency matched with patients by 5-year age groups, race, and telephone exchanges (area code plus 3-digit prefixes for cases from New York City) or county of residence (cases from New Jersey). In New Jersey, controls for Black patients were also recruited through outreach sources, such as health events [17].

Of patients eligible for inclusion, more than 95% signed a release form for their tumor tissue as part of the informed consent. Clinical and tumor characteristics, including the expression status of ER, progesterone receptor (PR), and human epidermal growth factor receptor 2 (HER2), were obtained from pathology reports. Formalin-fixed paraffin-embedded tissue specimens were used for tissue microarray construction that was guided by a specialized breast pathologist (T. K.) and included at least 2 cores, ranging in size from 0.6 to 1.2 mm, per patient. In total, samples from 770 cases included in tissue microarrays (TMAs) were available for laboratory assays. After immunostaining, patients with tumor tissue cores with insufficient cells (< 25 cells) for scoring were excluded, and 1 case and 1 control were excluded for missing BMI data, resulting in a final data set of 715 cases (574 [80%] Black and 141 [20%] White patients) and 1983 controls (1280 [64%] Black and 713 [36%] White women). The percentage of Black women in cases was higher than that in controls because WCHS focused on collecting tumor specimens in Black women in the later stage of the study. Among the cases, 644 had invasive breast cancer, and 71 had DCIS.

### Immunohistochemistry (IHC) and image analysis

We used p-mTOR to indicate mTOR pathway activation as the protein expression was positively correlated with BMI as well as key phosphorylated proteins in the upstream and downstream of the mTOR pathway, including p-AKT, p-p70S6K, and p-4EBP1 [14, 15]. Sections from TMAs were cut at 5 μm, placed on charged slides, and dried at 60 °C for 1 h. Slides were cooled to room temperature, placed in the Dako Omnis autostainer, where they were deparaffinized with Clearify, and rehydrated using graded alcohols. Flex Target Retrieval Solution, High pH (Agilent; catalog No. GV80411) was used for 30 min. Slides were incubated with p-mTOR antibody (Ser2448, clone 49F9, Cell Signaling; catalog No. 2976) for 40 min at a dilution of 1:75. Envision FLEX HRP-labeled polymer (Agilent; catalog No. DM842) was applied for 30 min. Diaminobenzidine (DAB; Agilent; catalog No. K3468), applied for 5 min, was used for chromogen visualization. Slides were counterstained with hematoxylin for 8 min and then placed in distilled water. After being removed from the autostainer, the sections were cleared and coverslipped.

**Fig. 1:**
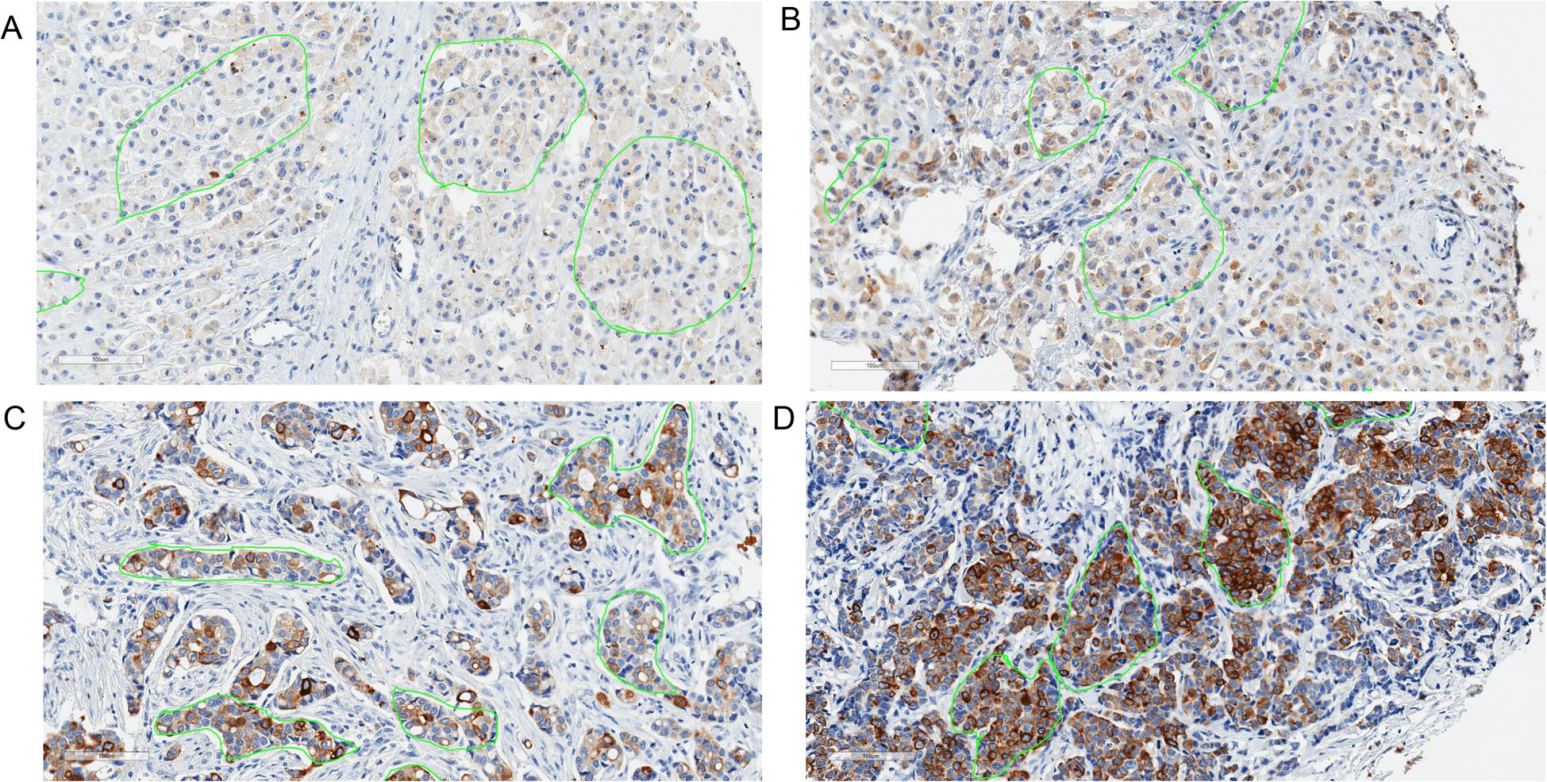
Images of p-mTOR Ser2448 immunohistochemistry staining in breast tumor tissue microarray cores. Tissues encircled in green indicate areas annotated for automatic scoring. **A** Histologic (H)-score = 3. **B** H-score = 24. **C** H-score = 92. **D** H-score = 183. Magnification = × 20; scale bar = 100 μM

Stained slides were digitally imaged at magnification × 20 using the Aperio ScanScope XT (Leica Biosystems) digital slide scanner system. Regions were identified and manually annotated to appropriately represent the heterogeneity of staining of each TMA core and to reduce irrelevant regions from image analysis calculations. The Aperio image analysis platform was used to develop quantitative image analysis algorithm macros for the quantification of IHC slides. A cytoplasmic algorithm was tailored to fine tune the cell feature detection using cellular, nuclear, and stain parameters, creating a specific algorithm macro for p-mTOR based on the cell compartment location of the target protein. The cytoplasmic algorithm analyzed DAB staining intensity and the percentage of cells containing stain within the cytoplasm compartment. The algorithm results provided total number of cells and percentage per scoring class (0, none; 1+, partial or weak; 2+, moderate; or 3+, strong). A histologic score (H-score) at the core level was calculated by the formula [1 × (% cells 1+) + 2 × (% cells 2+) + 3 × (% cells 3+)] × 100 [18]. The core-level data were collapsed into case-level data using a cellularity-weighted approach [19]. Figure 1 shows representative images of p-mTOR IHC staining.

### Epidemiologic and anthropometric data collection

Data on demographic characteristics, history of benign breast disease, reproductive and menstrual histories, family history of breast cancer in a first-degree relative, oral contraceptive use, and history of diabetes and its treatment were obtained by in-person interviews [20]. Anthropometric measurements were obtained by trained staff during the in-person interviews using a standardized protocol previously described [20]. WC was measured at the umbilicus, and hip circumference was measured at the maximum extension of the buttocks. Fat mass and percent body fat were measured by bioelectrical impedance analysis using a Tanita TBF-300A total body composition analyzer. Fat mass index was calculated as fat mass in kilograms divided by the square of height in meters.

### Statistical analysis

Breast cancer cases with p-mTOR overexpressed tumors were defined as having p-mTOR expression levels above the 75th percentile level (i.e., approximate H-score = 80), and cases with p-mTOR negative/low tumors were those having expression levels below the 75th percentile. Because there is no standard definition for p-mTOR overexpression, we examined 2 additional cutoff points of p-mTOR expression levels: 25th percentile (H-score = 1) and 50th percentile (H-score = 20) for defining p-mTOR overexpressed tumors. Demographics and body fatness measurements were compared among p-mTOR overexpressed cases vs. controls and p-mTOR negative/low cases vs. controls using *t* tests for continuous variables and *χ*^2^ tests for categorical variables. We performed polytomous (as known as multinomial) logistic regression to estimate odds ratios (ORs) and 95% confidence intervals (CIs) for p-mTOR overexpressed and for p-mTOR negative/low breast cancer in relation to the body fatness variables [21]. The models were adjusted for age, race, family history of breast cancer, age at menarche, parity, breastfeeding, menopausal status, oral contraceptive use, and history of diabetes (defined as using any medications or injections for diabetes). BMI was classified as < 25.0 (normal weight), 25.0–29.9 (overweight), 30.0–34.9 (class I obesity), and ≥ 35.0 kg/m^2^ (class II/III obesity) [22]. WC, WHR, and percent body fat were classified into quartiles based on their distributions in controls. Because body fatness variables were positively correlated with each other at various levels, for example, BMI was highly associated with WC, percent body fat, and fat mass index (*r* > 0.80), and WHR was modestly correlated with BMI, percent body fat, and fat mass index (*r* = 0.35 to 0.40; Supplemental Table 1), these variables were not mutually adjusted to prevent multicollinearity. Trend tests were performed by using the median values of each category of the body fatness variables as a continuous variable in the regression models. The differences in the associations of body fatness with breast cancer risk by the p-mTOR expression status were assessed by tests for heterogeneity with a null hypothesis that the relationship between body fatness and p-mTOR overexpressed tumors was not different from that for p-mTOR negative/low tumors, i.e., disease subtype heterogeneity hypothesis [23]. For stratified analyses, we performed separate regression models for ER+ and ER– tumors. We also stratified the associations by menopausal status and by race as secondary analyses because breast cancer etiology related to obesity may differ by these factors [24]. Two sets of sensitivity analyses were performed to evaluate the associations for invasive breast cancer cases vs. controls and for ER+/PR+ and ER–/PR– tumor subtypes vs. controls. All tests were two-sided, and *P* < .05 was considered statistically significant. Statistical analyses were performed using SAS, version 9.4 (SAS Institute Inc).

## Results

We compared the ORs of p-mTOR overexpressed tumors defined by the 3 cutoff points of p-mTOR expression (25th, 50th, and 75th percentiles) in association with body size (Supplemental Table 2). In general, the associations were stronger when 75th percentile was used as the cutoff compared to those with lower cutoffs. For example, the ORs for quartile (Q)4 vs. Q1 WC were 1.23 (95% CI = 0.90 to 1.69) for the 25th percentile, 1.39 (95% CI = 0.95 to 2.05) for the 50th percentile, and 1.99 (95% CI = 1.12 to 3.50) for the 75th percentile levels of p-mTOR expression used to define p-mTOR overexpressed tumors.

Table 1 gives the selected characteristics, including body fatness, of cases with p-mTOR overexpressed tumors, cases with p-mTOR negative/low tumors, and controls. Patients who had p-mTOR overexpressed tumors were more likely than controls to have higher BMI, larger WC, higher WHR, and higher levels of body fat (all *P* < 0.05). Patients who had p-mTOR negative/low tumors were more likely than controls to have BMI ≥ 30, larger WC, and higher WHR, but to a lesser extent compared to the differences between the cases with p-mTOR overexpressed tumors and controls. Compared with patients having p-mTOR negative/low tumors, patients with p-mTOR overexpressed tumors had higher BMI, larger WC, and more body fat, and their tumors were more likely to be ER+ (87.0% vs. 59.5%).

**Table 1:** Selected characteristics of demographics, body fatness, and breast tumors of the cases and controls

Characteristic	Cases with p-mTOR overexpressed tumors (*n* = 192)^a^	Cases with p-mTOR negative/low tumors (*n* = 523)	Controls (*n* = 1983)	*P* value (p-mTOR overexpressed vs controls)^b^	*P* value (p-mTOR negative/low vs controls)^b^	*P* value (p-mTOR overexpressed vs p-mTOR negative/low)^b^
Age, mean (SD), years	53.8 (10.7)	52.5 (11.1)	48.6 (9.4)	< 0.001	< 0.001	0.16
Race, no. (%)						
Black	159 (82.8)	415 (79.4 )	1270 (64.0)	< 0.001	< 0.001	0.30
White	33 (17.2)	108 (20.6)	713 (36.0)			
Menopausal status, no. (%)						
Premenopausal	86 (44.8)	241 (46.1)	982 (49.5)	0.21	0.16	0.76
Postmenopausal	106 (55.2)	282 (53.9)	1001 (50.5)			
BMI, kg/m^2^, no. (%)						
< 25.0	31 (16.2)	121 (23.1)	553 (27.9)	0.001	0.010	0.033
25.0–29.9	49 (25.5)	150 (28.7)	534 (26.9)			
30.0–34.9	51 (26.6)	135 (25.8)	400 (20.2)			
≥ 35.0	61 (31.8)	117 (22.4)	496 (25.0)			
WC, inches, no.	190	513	1965			
Mean (SD)	102.8 (17.9)	98.2 (14.7)	96.7 (17.1)	< 0.001	0.07	0.002
WHR, no.	190	513	1965			
Mean (SD)	0.88 (0.08)	0.88 (0.09)	0.86 (0.09)	< 0.001	< 0.001	0.29
Percentage body fat, no.	182	496	1893			
Mean (SD), %	41.1 (7.6)	39.2 (7.6)	38.8 (8.8)	< 0.001	0.22	0.006
Fat mass index, no.	181	495	1868			
Mean (SD), kg/m^2^	13.8 (5.1)	12.3 (4.7)	12.3 (5.7)	< 0.001	0.78	< 0.001
ER status, no. (%)						
Positive	167 (87.0)	310 (59.5)	–	–	–	< 0.001
Negative	25 (13.0)	211 (40.5)	–	–	–	
PR status, no. (%)						
Positive	163 (85.8)	308 (59.2)	–	–	–	< 0.001
Negative	27 (14.2)	213 (40.9)	–	–	–	
HER2 status, no. (%)						
Positive	31 (17.2)	111 (22.0)	–	–	–	0.18
Negative	149 (82.8)	394 (78.0)	–	–	–	

*Abbreviations*: *BMI* body mass index, *ER* estrogen receptor, *HER2* human epidermal growth factor receptor 2, *PR* progesterone receptor, *SD* standard deviation, *WC* waist circumference, *WHR* waist-to-hip ratio

^a^p-mTOR overexpression defined as H-score ≥ 80 (the 75th percentile); p-mTOR negative/low expression defined as H-score < 80

^b^Estimated by *t* test for continuous variables and chi-square test for categorical variables

Results from multivariable models (Table 2) showed that cases with p-mTOR overexpressed tumors had a higher odds of being obese or very obese (BMI = 30.0–34.9 or ≥ 35.0) compared to controls, although the OR estimates were not significant. However, the association was different from that of cases with mTOR negative/low tumors compared to controls (*P*-heterogeneity = 0.011). There was an inverse association between BMI ≥ 35 and p-mTOR negative/low tumors (OR = 0.70, 95% CI = 0.52–0.95; P-trend = 0.017). For the measurement of central adiposity, cases with p-mTOR overexpressed tumors had a higher odds of being at the Q3 (OR = 2.52, 95% CI = 1.46 to 4.34) and Q4 (OR = 1.99, 95% CI = 1.12 to 3.50) of WC compared to controls. Similarly, cases with p-mTOR overexpressed tumors had a higher odds of being at the Q3 (OR = 1.82, 95% CI = 1.11 to 2.98) and Q4 (OR = 1.81, 95% CI = 1.11 to 2.98) of WHR compared to controls. These associations of WC and WHR were not observed in cases with p-mTOR negative/low tumors compared to controls, although the associations did not differ by tumor p-mTOR status (*P*-heterogeneity = 0.27 and 0.48, respectively). In the subgroups, the association of BMI with p-mTOR overexpressed breast cancer appeared stronger in postmenopausal women (*P*-trend = 0.09) than premenopausal women (*P*-trend = 0.92) (Supplemental Table 3). However, the associations of WC and WHR with p-mTOR overexpressed breast cancer were similar between premenopausal and postmenopausal women. In addition, the associations of WC and WHR with p-mTOR overexpressed tumors were observed in Black women, but not in White women (Supplemental Table 4). In the sensitivity analysis restricted to invasive breast cancer cases vs. controls, the overall associations of body size variables with p-mTOR overexpressed and p-mTOR negative/low breast cancer remained the same as those in all cases vs. controls (Supplemental Table 5).

**Table 2:** Odds ratios of breast cancer risk in relation to p-mTOR expression associated with body size measurements

Body size measurement	p-mTOR overexpressed cases vs controls	p-mTOR negative/low cases vs controls	P-heterogeneity
	OR (95% CI)	OR (95% CI)	
*BMI,* kg/m^2^			
No. of cases/no. of controls	189/1973	522/1973	
< 25	1.00 [reference]	1.00 [reference]	
25–29.99	1.22 (0.75–1.97)	1.02 (0.77–1.35)	
30–34.99	1.52 (0.92–2.47)	1.11 (0.83–4.19)	
≥ 35	1.31 (0.80–2.13)	0.70 (0.52–0.95)	0.011
P-trend	0.31	0.017	
*WC*			
No. of cases/no. of controls	187/1955	512/1955	
Q1	1.00 [reference]	1.00 [reference]	
Q2	1.67 (0.74–2.96)	1.10 (0.81–1.50)	
Q3	2.52 (1.46–4.34)	1.24 (0.91–1.67)	
Q4	1.99 (1.12–3.50)	0.94 (0.69–1.30)	0.27
P-trend	0.029	0.64	
*WHR*			
No. of cases/no. of controls	187/1955	512/1955	
Q1	1.00 [reference]	1.00 [reference]	
Q2	1.21 (0.71–2.06)	1.30 (0.96–1.77)	
Q3	1.82 (1.11–2.98)	1.32 (0.97–1.80)	
Q4	1.81 (1.11–2.98)	1.34 (0.98–1.83)	0.48
P-trend	0.009	0.10	

*Abbreviations*: *BMI* body mass index, *OR* odds ratio, *Q* quartile, *WC* waist circumference, *WHR* waist-to-hip ratio

Models adjusted for age, race, family history of breast cancer, menopausal status, age of menarche, parity, history of breastfeeding, oral contraceptive use, and history of diabetes

Waist circumference (WC) quartile cutoffs: 83.7, 94.7, and 107.4 inches

Waist-to-hip ratio (WHR) quartile cutoffs: 0.804, 0.855, and 0.911

P-heterogeneity: tests for differences in the associations of body fatness measurements between p-mTOR overexpressed tumors and p-mTOR negative/low tumors

In the analyses stratified by tumor ER status (Table 3), the difference in the association of BMI with breast cancer risk by p-mTOR expression status was significant among women with ER+ tumors (*P*-heterogeneity = 0.015), but not those with ER– tumors. Also among women with ER+ tumors, larger (Q3 and Q4) vs. smaller (Q1) of WC and WHR were associated with increased risk of p-mTOR overexpressed tumors. Data among women with ER– tumors also suggest such associations, although the estimates were significant only for women in Q3 (OR = 4.70, 95% CI = 1.00 to 22.07 for WC and OR = 5.35, 95% CI = 1.16 to 24.71 for WHR). Similar results were observed for ER+/PR+ and ER–/PR– tumors (Supplemental Table 6).

**Table 3:** Odds ratios of breast cancer risk in relation to p-mTOR expression associated with body size measurements by tumor ER status

Body size measurement		ER+ tumors			ER– tumors	
	p-mTOR overexpressed cases vs controls	p-mTOR negative/low cases vs controls	P-heterogeneity	p-mTOR overexpressed cases vs controls	p-mTOR negative/low cases vs controls	P-heterogeneity
	OR (95% CI)	OR (95% CI)		OR (95% CI)	OR (95% CI)	
BMI, kg/m^2^						
No. of cases/no. of controls	164/1973	309/1973		25/1973	211/1973	
< 25	1.00 [reference]	1.00 [reference]		1.00 [reference]	1.00 [reference]	
25–29.99	1.29 (0.77–2.16)	1.02 (0.72–1.45)		0.83 (0.23–2.99)	1.00 (0.68–1.49)	
30–34.99	1.48 (0.87–2.52)	1.33 (0.92–1.91)		1.72 (0.54–5.48)	0.84 (0.54–1.31)	
≥ 35	1.39 (0.82–2.34)	0.75 (0.51–1.11)	0.015	0.90 (0.25–3.25)	0.61 (0.39–0.96)	0.09
P-trend	0.27	0.18		0.97	0.015	
*WC*						
No. of cases/no. of controls	162/1955	304/1955		25/1955	206/1955	
Q1	1.00 [reference]	1.00 [reference]		1.00 [reference]	1.00 [reference]	
Q2	1.65 (0.90–3.01)	1.09 (0.74–1.60)		1.81 (0.32–10.1)	1.09 (0.70–1.70)	
Q3	2.28 (1.28–4.06)	1.36 (0.93–1.98)		4.70 (1.00–22.07)	1.04 (0.57–1.63)	
Q4	1.93 (1.06–3.51)	1.01 (0.67–1.50)	0.26	2.60 (0.50–13.6)	0.83 (0.52–1.32)	0.55
P-trend	0.050	0.97		0.25	0.31	
*WHR*						
No. of cases/no. of controls	162/1955	304/1955		25/1955	206/1955	
Q1	1.00 [reference]	1.00 [reference]		1.00 [reference]	1.00 [reference]	
Q2	1.17 (0.57–2.04)	1.34 (0.91–1.98)		1.58 (0.26–9.59)	1.34 (0.85–2.10)	
Q3	1.54 (0.91–2.60)	1.47 (1.00–2.15)		5.35 (1.16–24.71)	1.28 (0.80–2.03)	
Q4	1.69 (1.01–2.84)	1.37 (0.93–2.03)	0.36	3.42 (0.68–17.16)	1.60 (0.99–2.58)	0.89
P-trend	0.027	0.14		0.08	0.38	

*Abbreviations*: *BMI* body mass index, *ER* estrogen receptor, *OR* odds ratio, *Q* quartile, *WC* waist circumference, *WHR* waist-to-hip ratio

Models adjusted for age, race, family history of breast cancer, menopausal status, age of menarche, parity, history of breastfeeding, oral contraceptive use, and history of diabetes

Cases with p-mTOR overexpressed tumors had an increased odds of having higher percentage body fat and fat mass index compared to controls, and the associations tended to be inversed for cases with p-mTOR negative/low tumors compared to controls (both *P*-heterogeneity = 0.001; Table 4), although the individual OR estimates were not significant. These patterns were similar when stratified by tumor ER status (all *P*-heterogeneity < 0.05; Table 5).

**Table 4:** Odds ratios of breast cancer risk in relation to p-mTOR expression associated with body composition measurements

Body composition measurement	p-mTOR overexpressed cases vs controls	p-mTOR negative/low cases vs controls	P-heterogeneity
	OR (95% CI)	OR (95% CI)	
*Percent body fat*			
No. of cases/no. of controls	179/1884	495/1884	
Q1	1.00 [reference]	1.00 [reference]	
Q2	1.26 (0.74–2.14)	1.29 (0.95–1.74)	
Q3	1.55 (0.93–2.58)	0.98 (0.72–1.33)	
Q4	1.31 (0.77–2.22)	0.71 (0.51–0.99)	0.001
P-trend	0.27	0.015	
*Fat mass index*			
No. of cases/no. of controls	179/1859	494/1859	
Q1	1.00 [reference]	1.00 [reference]	
Q2	1.22 (0.72–2.08)	1.39 (1.03–1.88)	
Q3	1.34 (0.79–2.26)	1.08 (0.79–1.49)	
Q4	1.42 (0.84–2.39)	0.77 (0.55–1.07)	0.001
P-trend	0.21	0.008	

*Abbreviations*: *OR* odds ratio, *Q* quartile

Models adjusted for age, race, family history of breast cancer, menopausal status, age of menarche, parity, history of breastfeeding, oral contraceptive use, and history of diabetes

Percent body fat quartile cutoffs: 32.7%, 39.7%, and 45.3%

Fat mass index cutoffs: 8.04, 11.67, and 15.63 kg/m^2^

**Table 5:** Odds ratios of breast cancer risk in relation to p-mTOR expression associated with body composition measurements by tumor ER status

Body composition measurement		ER+ tumors			ER– tumors	
	p-mTOR overexpressed cases vs controls	p-mTOR negative/low cases vs controls	P-heterogeneity	p-mTOR overexpressed cases vs controls	p-mTOR negative/low cases vs controls	P-heterogeneity
	OR (95% CI)	OR (95% CI)		OR (95% CI)	OR (95% CI)	
*Percent body fat*						
No. of cases/no. of controls	156/1884	295/1884		23/1884	198/1884	
Q1	1.00 [reference]	1.00 [reference]		1.00 [reference]	1.00 [reference]	
Q2	1.29 (0.73–2.28)	1.10 (0.76–1.60)		1.08 (0.28–4.20)	1.53 (0.99–2.36)	
Q3	1.61 (0.93–2.78)	1.06 (0.71–1.50)		1.20 (0.31–4.58)	0.84 (0.52–1.36)	
Q4	1.31 (0.74–2.32)	0.67 (0.44–1.01)	0.037	1.34 (0.36–4.95)	0.73 (0.45–1.19)	0.003
P-trend	0.31	0.06		0.64	0.040	
*Fat mass index*						
No. of cases/no. of controls	155/1859	294/1859		23/1859	198/1859	
Q1	1.00 [reference]	1.00 [Reference]		1.00 [Reference]	1.00 [reference]	
Q2	1.26 (0.71–2.23)	1.24 (0.85–1.80)		1.06 (0.27–4.41)	1.59 (1.02–2.47)	
Q3	1.36 (0.77–2.38)	1.19 (0.81–1.74)		1.23 (0.33–4.61)	0.89 (0.55–1.45)	
Q4	1.48 (0.84–2.60)	0.72 (0.47–1.10)	0.011	1.09 (0.29–4.20)	0.79 (0.48–1.30)	0.004
P-trend	0.19	0.040		0.90	0.031	

*Abbreviations*: *ER* estrogen receptor, *OR* odds ratio, *Q* quartile

Models adjusted for age, race, family history of breast cancer, menopausal status, age of menarche, parity, history of breastfeeding, oral contraceptive use, history of diabetes

## Discussion

Our data suggested that breast cancer cases with overexpressed p-mTOR tumors had higher levels of body fatness than controls, and the association was not observed in cases with negative/low p-mTOR tumors compared to controls. Significant differences between the associations were observed for BMI, percent body fat, and fat mass index. Also, cases with p-mTOR overexpressed tumors had approximately twofold increased odds of having higher (Q3 and Q4) WC and WHR compared to controls. Our findings suggest that mTOR pathway activation indicated by p-mTOR expression may be relevant to the association between body fatness and breast cancer risk. To our knowledge, this study is among the first to examine the associations between body fatness measurements and the risk of breast tumors with p-mTOR overexpression.

Our stratified analyses on tumor ER status and menopausal status showed results consistent with our current understanding of obesity in association with breast cancer risk. If mTOR pathway activation is a mechanism by which body fatness influences breast cancer risk, the association of body fatness in relation to mTOR activation should be observed in postmenopausal women with ER+ tumors for obesity defined by high BMI, as the epidemiologic evidence indicates [2–5]. In our data, we observed a trend that higher vs. lower BMI was associated with p-mTOR overexpressed tumors, and the association was mainly in ER+ tumors and in postmenopausal women. For central obesity, our findings are also consistent with the AMBER Consortium findings that higher vs. lower WHR was associated with ER+ or ER– tumors in either premenopausal or postmenopausal women [5]. However, for ER+ tumors, it may be difficult to tease out the independent influence of body fatness in mTOR from the hormonal influence of estrogen synthesized by aromatase in the adipose tissue. The mTOR pathway cross-talks with the estrogen signaling pathway and can be promoted by estrogens [25], and the protein expression of mTOR and p-mTOR was higher in ER+ than ER– tumors in our study population [14]. On the contrary, for ER– tumors, our findings are considered less confounded by ER. We found indications of associations of WC and WHR with p-mTOR overexpressed tumors among women with ER– tumors. The OR estimates were imprecise because the number of cases with p-mTOR-overexpressed and ER– tumors was small (*N* = 25). Our findings require replication with a larger sample of ER– tumors. If confirmed, our findings suggest that targeting the mTOR pathway using pharmacological approaches, such as metformin [26, 27] and other mTOR inhibitors, may be able to prevent a subset of ER+ and ER– tumors.

We observed an inverse association between BMI ≥ 35 vs. < 25 and p-mTOR-negative/low breast cancer overall and among women with ER– tumors. The association was comparable for both premenopausal and postmenopausal women (data not shown). Similarly, in the WCHS and AMBER Consortium, BMI ≥ 30 or 35 was associated with a lower risk of ER– or triple-negative breast cancer in postmenopausal women [5, 20]. The explanation for the association is unclear. We showed that the association might exist only for tumors with p-mTOR negative/low expression but not tumors with p-mTOR overexpression. Thus, our findings suggest that mechanisms other than mTOR may explain the inverse association between BMI and ER– breast cancer.

We acknowledge that the body fatness measurement in this study may not accurately reflect the specific adiposity compartments that are most relevant to mTOR pathway activation and breast cancer etiology. The body composition of participants was assessed by bioelectrical impedance analysis, and the method may have larger errors among those with lower vs. higher percent of body fat [28]. BMI is not an accurate measure of body fatness as body weight involves the weights of muscle and other body compartments [29]. WC and WHR are strongly correlated with abdominal adiposity [30]. In abdominal adiposity, visceral adipocytes may more strongly influence insulin sensitivity and organ tissues than subcutaneous adipocytes do [30]. Future research using body fat measurements based on imaging [31, 32] is warranted to reveal the role of mTOR pathway activation in association with specific adipose tissue compartments.

The strengths of our study include rigorous and quantitative measurements of protein expression in breast tumors and multiple measurements of body fatness. We manually annotated the stained tissue to minimize influences from other tissue components, such as the stroma, and performed automated imaging analysis to derive objective scores. The continuous H-score has the flexibility to modify cutoffs for defining p-mTOR overexpression. In addition, body fatness was measured by trained staff who used a standardized protocol. The sample included a relatively large number of Black women, a population with high prevalence of obesity in general and central obesity [24] as well as ER– tumors. Also, we were able to adjust for a wide range of reproductive and hormonal risk factors in our statistical analyses.

Currently, there is no standard approach to define the activation of mTOR pathway; however, correlating with a phenotype that is biologically relevant appears to be a feasible approach. In a panel of four mTOR pathway makers (mTOR, p-mTOR, p-AKT, and p-P70S6K) that we evaluated in patients with breast cancer, p-mTOR has the strongest association in relation to body fatness [14]. In addition, protein expression levels of p-MTOR were modestly correlated with p-AKT and p-p70S6K (*r* = 0.26–0.31) and highly correlated with total phosphoprotein levels (*r* = 0.71), which is a summation of the three phosphoproteins (Supplemental Table 7). Also, in breast cancer samples from The Cancer Genome Atlas (TCGA), p-mTOR expression was also correlated with p-AKT, p-p70S6K, and a mTOR signature (*r* = 0.21–0.45) that was derived from expression levels of seven phosphoproteins in the pathway (Supplemental Table 7). Using a larger panel of phosphoproteins in the mTOR pathway to evaluate the associations is warranted for future studies.

Several other limitations of our study should be noted. First, as an inherent bias of case-control studies, body fatness was assessed after breast cancer diagnosis among cases. The observed associations might have been affected if the participants had changes in weight or body composition because of breast cancer development and treatment [33]. Studies with a prospective design are warranted to confirm our observations. Also, our study design limits the ability to draw an inference because tissue samples were unavailable among the controls to assess the p-mTOR protein expression. Reasons other than body fatness or excessive energy intake, such as mutations, may increase p-mTOR expression because tumor growth is autonomous. Second, there is no standard cutoff or phenotype that can be used as a reference for defining p-mTOR overexpression. The cutoff we proposed may not be applicable to other populations. Because protein phosphorylation is part of the normal physiology of mTOR signaling, we believe it is reasonable to set a higher expression level of p-mTOR as a cutoff for defining overexpression. However, future research is needed to determine the cutoff of p-mTOR expression that leads to physiologic and pathological changes. In addition, it is unknown whether p-mTOR expression changes associated with body fatness were long-term or short-term. Third, the investigation of different measurements of body fatness resulted in multiple comparisons, potentially leading to false-positive results. This concern was minimal as we performed planned analyses with a priori hypotheses. Lastly, the percentage of Black women in cases was higher than that in controls due to the study design. We were unable to conclude race-specific risks, particularly in White women, because of the low number of White women in p-mTOR-overexpressed cases.

## Conclusions

In this case-control study with a large proportion of Black women, we compared several body fatness measurements between cases with p-mTOR overexpressed tumors or p-mTOR negative/low tumors with non-cancer controls. We found that higher vs. lower levels of body fatness tended to be associated with p-mTOR overexpressed tumors, but not p-mTOR negative/low tumors. The associations stratified by tumor ER status and menopausal status are largely consistent with epidemiologic findings on body fatness and breast cancer risk. Our findings suggest that mTOR and its related signaling pathway is potentially an underlying mechanism by which body fatness influences breast cancer risk. The strength of the associations was modest (OR = 1.5–2.0), suggesting that the influence of obesity fatness on breast cancer risk is likely through multiple mechanisms. Our findings warrant further confirmation with studies of larger sample size, as statistical power in the current study may not be sufficient in detecting the heterogeneity of the associations between ER subtypes and estimating the risks within a subtype. A prospective study design is also necessary to confirm the associations.

## Supplementary Information

**Additional file 1: Supplemental Table 1.** Pearson correlation coefficients of the body fatness measurements. **Supplemental Table 2.** Odds ratios for p-mTOR-overexpressed breast cancer, with p-mTOR overexpression defined as above the 25th, 50th, and 75th percentile of the H-score, in association with BMI, WC, and WHR. **Supplemental Table 3.** Odds ratios for p-mTOR-overexpressed breast cancer in association with body size measurements by menopausal status. **Supplemental Table 4.** Odds ratios for p-mTOR-overexpressed breast cancer in association with body size measurements by race. **Supplement Table 5.** Odds ratios of breast cancer risk in relation to p-mTOR expression associated with body size measurements among invasive breast cancer cases and controls. **Supplemental Table 6.** Odds ratios of breast cancer risk in relation to p-mTOR expression associated with body size measurements in ER+/PR+ and ER–/PR– tumors. **Supplemental Table 7.** Pearson correlation coefficients (r) for phosphoprotein expression levels.

## Data Availability

The data supporting the findings of this study are not publicly available in order to protect patient privacy. The data will be made available to authorized researchers who have IRB approval.
