# Supplementary material for: Body fatness and breast cancer risk in relation to phosphorylated mTOR expression in a sample of predominately Black women
Source: Breast Cancer Res. 2021 Jul 30;23:77. doi: 10.1186/s13058-021-01458-z (PMC8325192; doi:10.1186/s13058-021-01458-z)
Supplement: Supplementary file 1 — Additional file 1: Supplemental Table 1. Pearson correlation coefficients of the body fatness measurements. Supplemental Table 2. Odds ratios for p-mTOR-overexpressed breast cancer, with p-mTOR overexpression defined as above the 25th, 50th, and 75th percentile of the H-score, in association with BMI, WC, and WHR. Supplemental Table 3. Odds ratios for p-mTOR-overexpressed breast cancer in association with body size measurements by menopausal status. Supplemental Table 4. Odds ratios for p-mTOR-overexpressed breast cancer in association with body size measurements by race. Supplement Table 5. Odds ratios of breast cancer risk in relation to p-mTOR expression associated with body size measurements among invasive breast cancer cases and controls. Supplemental Table 6. Odds ratios of breast cancer risk in relation to p-mTOR expression associated with body size measurements in ER+/PR+ and ER–/PR– tumors. Supplemental Table 7. Pearson correlation coefficients (r) for phosphoprotein expression levels. [file 13058_2021_1458_MOESM1_ESM.docx]

Supplemental Table 1. Pearson correlation coefficients of the body fatness measurements

| Measure | BMI | WC | WHR | Percent body fat | Fat mass index |
| --- | --- | --- | --- | --- | --- |
| BMI | 1.00 | - | - | - | - |
| WC | 0.90 | 1.00 | - | - | - |
| WHR | 0.35 | 0.60 | 1.00 | - | - |
| Percentage of body fat | 0.82 | 0.83 | 0.40 | 1.00 | - |
| Fat mass index | 0.97 | 0.90 | 0.36 | 0.91 | 1.00 |

Abbreviations: BMI, body mass index; WC, waist circumference; WHR, waist-to-hip ratio.

All *P* < .001

Supplemental Table 2: Odds ratios for p-mTOR-overexpressed breast cancer, with p-mTOR overexpression defined as above the 25^th^, 50^th^, and 75^th^ percentile of the H-score, in association with BMI, WC, and WHR

| Measure | p-mTOR expression cutoff | | |
| --- | --- | --- | --- |
|  | 25^th^ percentile (H-score ≥1) | 50^th^ percentile (H-score ≥20) | 75^th^ percentile (H-score ≥80) |
|  | OR (95% CI) | OR (95% CI) | OR (95% CI) |
| *BMI*, kg/m^2^ |  |  |  |
| No. of cases/No. of controls | 564/1973 | 372/1973 | 189/1973 |
| <25 | 1.00 [Reference] | 1.00 [Reference] | 1.00 [Reference] |
| 25-29.99 | 1.12 (0.64-1.48) | 0.96 (0.68-1.36) | 1.22 (0.75-1.97) |
| 30-34.99 | 1.28 (0.95-1.72) | 1.36 (0.96-1.93) | 1.52 (0.92-2.47) |
| ≥35 | 0.97 (0.72-1.31) | 0.97 (0.68-1.38) | 1.31 (0.80-2.13) |
| *WC* |  |  |  |
| No. of cases/No. of controls | 555/1955 | 364/1955 | 187/1955 |
| Q1 | 1.00 [Reference] | 1.00 [Reference] | 1.00 [Reference] |
| Q2 | 1.20 (0.0.88-1.64) | 1.12 (0.76-1.66) | 1.67 (0.74-2.96) |
| Q3 | 1.59 (1.17-2.15) | 1.75 (1.20-2.53) | 2.52 (1.46-4.34) |
| Q4 | 1.23 (0.90-1.69) | 1.39 (0.95-2.05) | 1.99 (1.12-3.50) |
| *WHR* |  |  |  |
| No. of cases/No. of controls | 555/1955 | 364/1955 | 187/1955 |
| Q1 | 1.00 [Reference] | 1.00 [Reference] | 1.00 [Reference] |
| Q2 | 1.27 (0.94-1.73) | 1.46 (1.00-2.15) | 1.21 (0.71-2.06) |
| Q3 | 1.44 (1.07-1.95) | 1.63 (1.19-2.36) | 1.82 (1.11-2.98) |
| Q4 | 1.45 (1.07-1.96) | 1.80 (1.24-2.60) | 1.81 (1.11-2.98) |

Abbreviations: BMI, body mass index; Q, quartile; WC, waist circumference; WHR, waist-to-hip ratio.

Models adjusted for age, race, family history of breast cancer, menopausal status, age of menarche, parity, history of breastfeeding, oral contraceptive use, and history of diabetes.

Supplemental Table 3: Odds ratios for p-mTOR-overexpressed breast cancer in association with body size measurements by menopausal status

| Body size measurement | p-mTOR-overexpressed cases vs. controls | |
| --- | --- | --- |
|  | Premenopausal women | Postmenopausal women |
|  | OR (95% CI) | OR (95% CI) |
| *BMI*, kg/m^2^ |  |  |
| No. of cases/No. of controls | 85/978 | 104/995 |
| <25 | 1.00 [Reference] | 1.00 [Reference] |
| 25-29.99 | 1.30 (0.69-2.47) | 1.18 (0.56-2.49) |
| 30-34.99 | 1.45 (0.73-2.88) | 1.68 (0.81-3.47) |
| ≥35 | 1.00 (0.49-2.04) | 1.73 (0.85-3.55) |
| P-trend | 0.92 | 0.09 |
|  |  |  |
| *WC* |  |  |
| No. of cases/No. of controls | 85/969 | 102/986 |
| Q1 | 1.00 [Reference] | 1.00 [Reference] |
| Q2 | 2.14 (1.01-4.54) | 1.22 (0.50-2.96) |
| Q3 | 2.67 (1.27-5.62) | 2.32 (1.03-5.22) |
| Q4 | 2.03 (0.91-4.51) | 1.94 (0.85-4.44) |
| P-trend | 0.12 | 0.08 |
|  |  |  |
| *WHR* |  |  |
| No. of cases/No. of controls | 85/969 | 102/986 |
| Q1 | 1.00 [Reference] | 1.00 [Reference] |
| Q2 | 0.96 (0.48-1.94) | 2.00 (0.81-4.97) |
| Q3 | 1.49 (0.79-2.84) | 2.95 (1.25-6.94) |
| Q4 | 1.57 (0.80-3.05) | 2.74 (1.18-6.36) |
| P-trend | 0.11 | 0.030 |

Abbreviations: BMI, body mass index; Q, quartile WC, waist circumference; WHR, waist-to-hip ratio.

Models adjusted for age, race, family history of breast cancer, age of menarche, parity, history of breastfeeding, oral contraceptive use, history of diabetes.

Supplemental Table 4: Odds ratios for p-mTOR-overexpressed breast cancer in association with body size measurements by race

| Body size measurement | p-mTOR-overexpressed cases vs. controls | |
| --- | --- | --- |
|  | OR (95% CI) | OR (95% CI) |
|  | Black women | White women |
| *BMI*, kg/m^2^ |  |  |
| No. of cases/No. of controls | 157/1266 | 32/707 |
| <25 | 1.00 [Reference] | 1.00 [Reference] |
| 25–29.99 | 1.46 (0.80-2.66) | 0.73 (0.28-1.89) |
| 30–34.99 | 1.83 (1.01-3.31) | 0.80 (0.27-2.40) |
| ≥35 | 1.58 (0.88-2.84) | 0.62 (0.18-2.10) |
| P-trend | 0.21 | 0.45 |
|  |  |  |
| *WC* |  |  |
| No. of cases/No. of controls | 155/1256 | 32/699 |
| Q1 | 1.00 [Reference] | 1.00 [Reference] |
| Q2 | 1.60 (0.78-3.29) | 2.01 (0.76-5.28) |
| Q3 | 2.79 (1.42-5.38) | 1.46 (0.50-4.28) |
| Q4 | 2.15 (1.08-4.28) | 1.35 (0.40-4.50) |
| P-trend | 0.036 | 0.67 |
|  |  |  |
| *WHR* |  |  |
| No. of cases/No. of controls | 155/1256 | 32/699 |
| Q1 | 1.00 [Reference] | 1.00 [Reference] |
| Q2 | 1.73 (0.87-3.44) | 0.70 (0.27-1.83) |
| Q3 | 2.93 (1.55-5.52) | 0.36 (0.11-1.19) |
| Q4 | 2.53 (1.34-4.77) | 1.22 (0.44-3.39) |
| P-trend | 0.004 | 0.85 |

Abbreviations: BMI, body mass index; OR, odds ratio; Q, quartile; WC, waist circumference; WHR, waist-to-hip ratio.

Models adjusted for age, family history of breast cancer, menopausal status, age of menarche, parity, history of breastfeeding, oral contraceptive use, and history of diabetes.

Supplement Table 5: Odds ratios of breast cancer risk in relation to p-mTOR expression associated with body size measurements among *invasive breast cancer* cases and controls

| Body size measurement | p-mTOR overexpressed cases vs. controls | p-mTOR negative/low cases vs. controls | P-heterogeneity |
| --- | --- | --- | --- |
|  | OR (95% CI) | OR (95% CI) |  |
| *BMI,* kg/m^2^ |  |  |  |
| No. of cases/No. of controls | 161/1973 | 479/1973 |  |
| <25 | 1.00 [Reference] | 1.00 [Reference] | 0.050 |
| 25-29.99 | 1.34 (0.81-2,23) | 1.00 (0.75-1.33) |  |
| 30-34.99 | 1.43 (0.84-2.43) | 1.12 (0.82-1.51) |  |
| ≥35 | 1.43 (0.85-2.41) | 0.66 (0.48-0.91) |  |
| P-trend | 0.25 | 0.011 |  |
|  |  |  |  |
| *WC* |  |  |  |
| No. of cases/No. of controls | 159/1955 | 470/1955 |  |
| Q1 | 1.00 [Reference] | 1.00 [Reference] | 0.13 |
| Q2 | 1.79 (0.98-3.27) | 1.12 (0.82-1.55) |  |
| Q3 | 2.40 (1.34-4.28) | 1.27 (0.93-1.74) |  |
| Q4 | 2.10 (1.15-3.82) | 0.96 (0.69-1.33) |  |
| P-trend | 0.029 | 0.69 |  |
|  |  |  |  |
| *WHR* |  |  |  |
| No. of cases/No. of controls | 159/1955 | 470/1955 |  |
| Q1 | 1.00 [Reference] | 1.00 [Reference] | 0.49 |
| Q2 | 1.17 (0.67-2.06) | 1.22 (0.89-1.68) |  |
| Q3 | 1.74 (1.04-2.93) | 1.27 (0.93-1.74) |  |
| Q4 | 1.70 (1.01-2.87) | 1.24 (0.90-1.71) |  |
| P-trend | 0.025 | 0.23 |  |

Abbreviations: BMI, body mass index; OR, odds ratio; Q, quartile; WC, waist circumference, WHR, waist-to-hip ratio.

Models adjusted for age, race, family history of breast cancer, menopausal status, age of menarche, parity, history of breastfeeding, oral contraceptive use, and history of diabetes.

Waist circumference (WC) quartile cutoffs: 83.7, 94.7, and 107.4 inches

Waist-to-hip ratio (WHR) quartile cutoffs: 0.804, 0.855, and 0.911

P-heterogeneity: Tests for differences in the associations of body fatness measurements between p-mTOR overexpressed tumors and p-mTOR negative/low tumors

Supplemental Table 6: Odds ratios of breast cancer risk in relation to p-mTOR expression associated with body size measurements in ER+/PR+ and ER–/PR– tumors

| Body size measurement | ER+/PR+ tumors | | | ER–/PR– tumors | | |
| --- | --- | --- | --- | --- | --- | --- |
|  | p-mTOR overexpressed cases vs. controls | p-mTOR negative/low cases vs. controls | P-heterogeneity | p-mTOR overexpressed cases vs. controls | p-mTOR negative/low cases vs. controls | P-heterogeneity |
|  | OR (95% CI) | OR (95% CI) |  | OR (95% CI) | OR (95% CI) |  |
| *BMI, kg/m^2^* |  |  |  |  |  |  |
| No. of cases/No. of controls | 151/1973 | 261/1973 |  | 16/1973 | 166/1973 |  |
| <25 | 1.00 [Reference] | 1.00 [Reference] |  | 1.00 [Reference] | 1.00 [Reference] |  |
| 25-29.99 | 1.50 (0.78-2.59) | 1.19 (0.81-1.74) |  | 1.07 (0.21-4.70) | 0.93 (0.60-1.44) |  |
| 30-34.99 | 1.58 (0.90-2.79) | 1.51 (1.01-2.23) |  | 1.19 (0.27-5.35) | 0.76 (0.46-1.24) |  |
| ≥35 | 1.61 (0.92-2.80) | 0.76 (0.50-1.17) | 0.050 | 0.53 (0.10-2.91) | 0.62 (0.38-0.99) | 0.86 |
| P-trend | 0.16 | 0.17 |  | 0.42 | 0.032 |  |
|  |  |  |  |  |  |  |
| *WC* |  |  |  |  |  |  |
| No. of cases/No. of controls | 149/1955 | 256/1955 |  | 16/1955 | 161/1955 |  |
| Q1 | 1.00 [Reference] | 1.00 [Reference] |  | 1.00 [Reference] | 1.00 [Reference] |  |
| Q2 | 1.77 (0.94-3.34) | 1.03 (0.68-1.58) |  | 2.06 (0.21-20.38) | 1.06 (0.65-1.73) |  |
| Q3 | 2.29 (1.24-4.24) | 1.40 (0.94-2.10) |  | 4.33 (0.52-36.59) | 0.95 (0.58-1.56) |  |
| Q4 | 1.19 (1.17-4.09) | 0.96 (0.62-1.48) | 0.17 | 1.91 (0.20-18.64) | 0.80 (0.48-1.34) | 0.17 |
| P-trend | 0.024 | 0.93 |  | 0.74 | 0.29 |  |
|  |  |  |  |  |  |  |
| *WHR* |  |  |  |  |  |  |
| No. of cases/No. of controls | 149/1955 | 256/1955 |  | 16/1955 | 161/1955 |  |
| Q1 | 1.00 [Reference] | 1.00 [Reference] |  | 1.00 [Reference] | 1.00 [Reference] |  |
| Q2 | 1.10 (0.62-1.97) | 1.38 (0.92-2.09) |  | 0.93 (0.06-15.08) | 1.10 (0.67-1.81) |  |
| Q3 | 1.48 (0.87-2.55) | 1.28 (0.84-1.94) |  | 5.78 (0.70-47.76) | 1.09 (0.67-1.80) |  |
| Q4 | 1.64 (0.96-2.79) | 1.35 (0.89-2.04) | 0.69 | 3.52 (0.40-31.06) | 1.16 (0.71-1.90) | 0.26 |
| P-trend | 0.038 | 0.27 |  | 0.17 | 0.59 |  |

Abbreviations: BMI, body mass index; ER, estrogen receptor; OR, odds ratio; PR, progesterone receptor; Q, quartile; WC, waist circumference, WHR, waist-to-hip ratio.

Models adjusted for age, race, family history of breast cancer, menopausal status, age of menarche, parity, history of breastfeeding, oral contraceptive use, and history of diabetes.

Supplemental Table 7. Pearson correlation coefficients (r) for phosphoprotein expression levels

1. Women’s Circle of Health Study (N = 590 patients with invasive breast cancer)

|  | p-mTOR (S2448) | p-AKT (S473) | p-p70S6 (T398) | Total phosphoprotein |
| --- | --- | --- | --- | --- |
| p-mTOR (S2448) | 1.00 | - | - | - |
| p-AKT (S473) | 0.31 | 1.00 | - | - |
| p-p70S6K (T398) | 0.26 | 0.52 | 1.00 | - |
| Total phosphoprotein | 0.70 | 0.67 | 0.69 | 1.00 |

Note: Protein expression was assayed using immunohistochemistry and measured as H-scores. Total phosphoprotein level was the summation of H-scores from the three phosphoproteins. H-scores were log-transformed for Pearson correlation coefficients. P-values were <0.001 for all correlation coefficients. Detailed methods are published in npj Breast Cancer 6, 45 (2020). https://doi.org/10.1038/s41523-020-00187-4.

1. The Cancer Genome Atlas (N = 874 patients with invasive breast cancer)

|  | p-mTOR (S2448) | p-AKT (S473) | p-p70S6 (T389) | mTOR signature |
| --- | --- | --- | --- | --- |
| p-mTOR (S2448) | 1.00 | - | - | - |
| p-AKT (S473) | 0.31 | 1.00 | - | - |
| p-p70S6K (T389) | 0.21 | 0.27 | 1.00 | - |
| mTOR signature | 0.45 | 0.31 | 0.41 | 1.00 |

Note: Phosphoproteins were assayed by reverse phase protein array. mTOR pathway signature was defined as the sum of standardized phosphoprotein levels of p-mTOR (S2448), p-4EBP1 (S65, T37/T46, and T70), p-P70S6K (T389), and p-S6 (S235/S236 and S240/S244). P-values were <0.001 for all correlation coefficients. Data are available in Zhang et al., 2017, Cancer Cell 31, 820–832.
